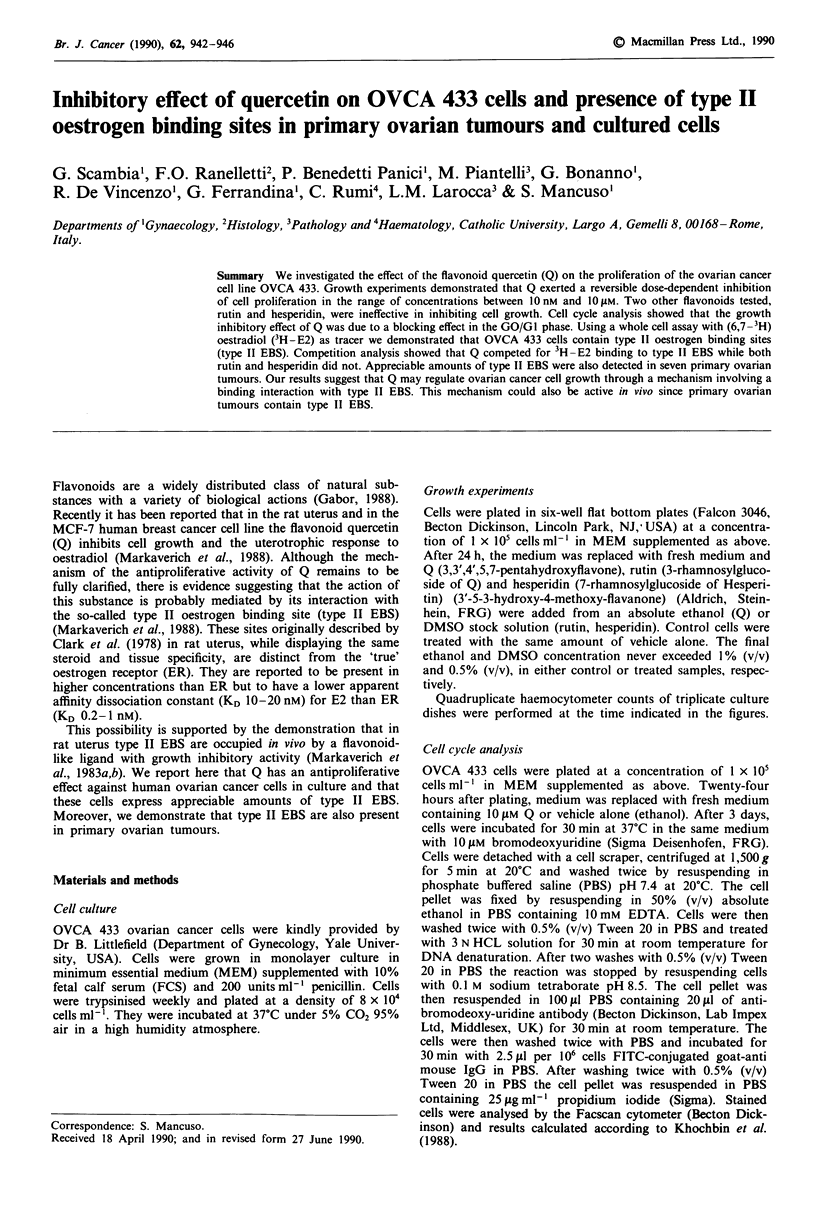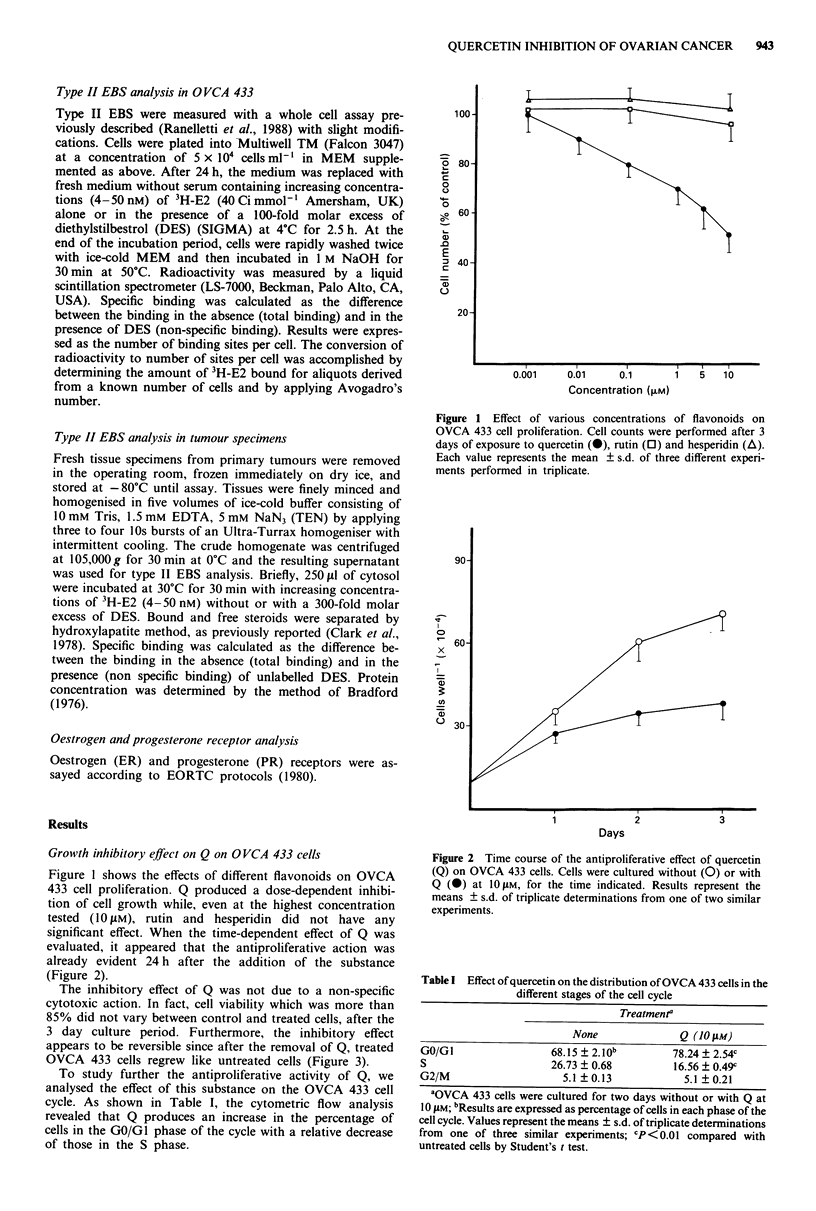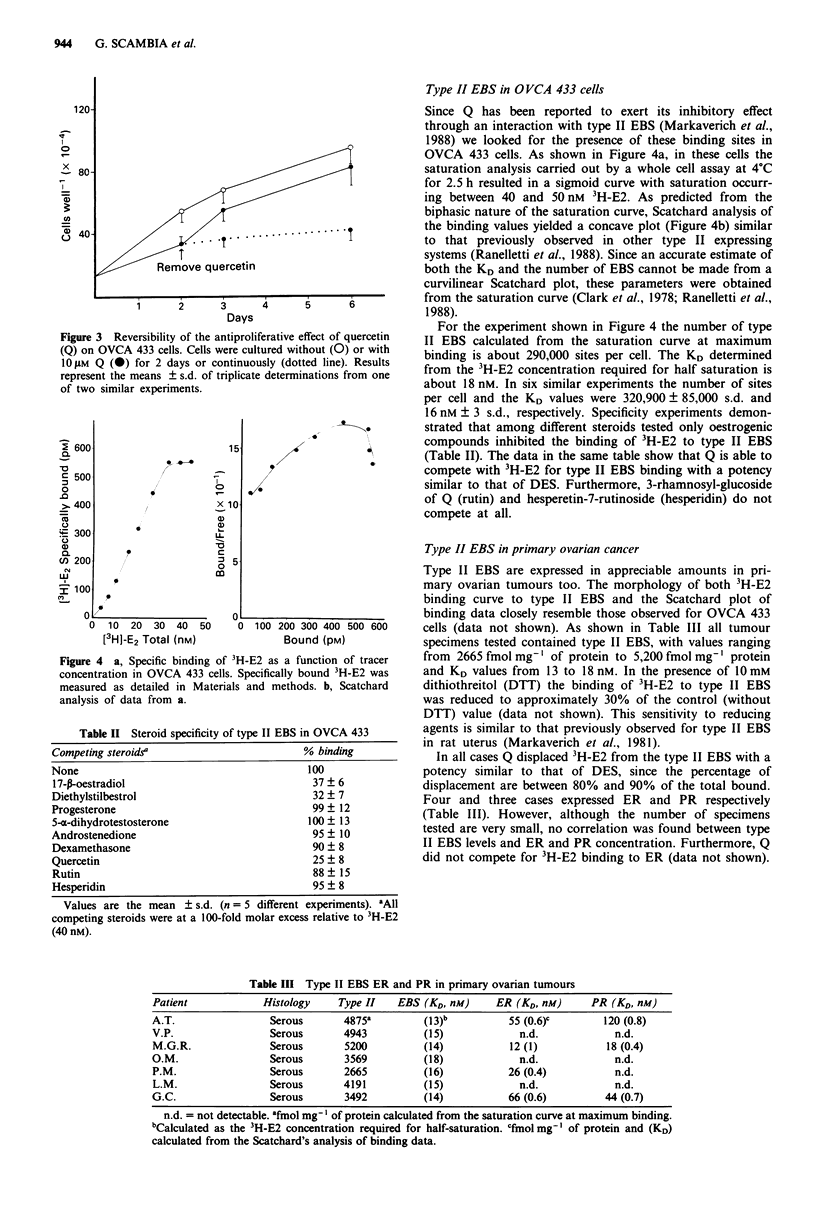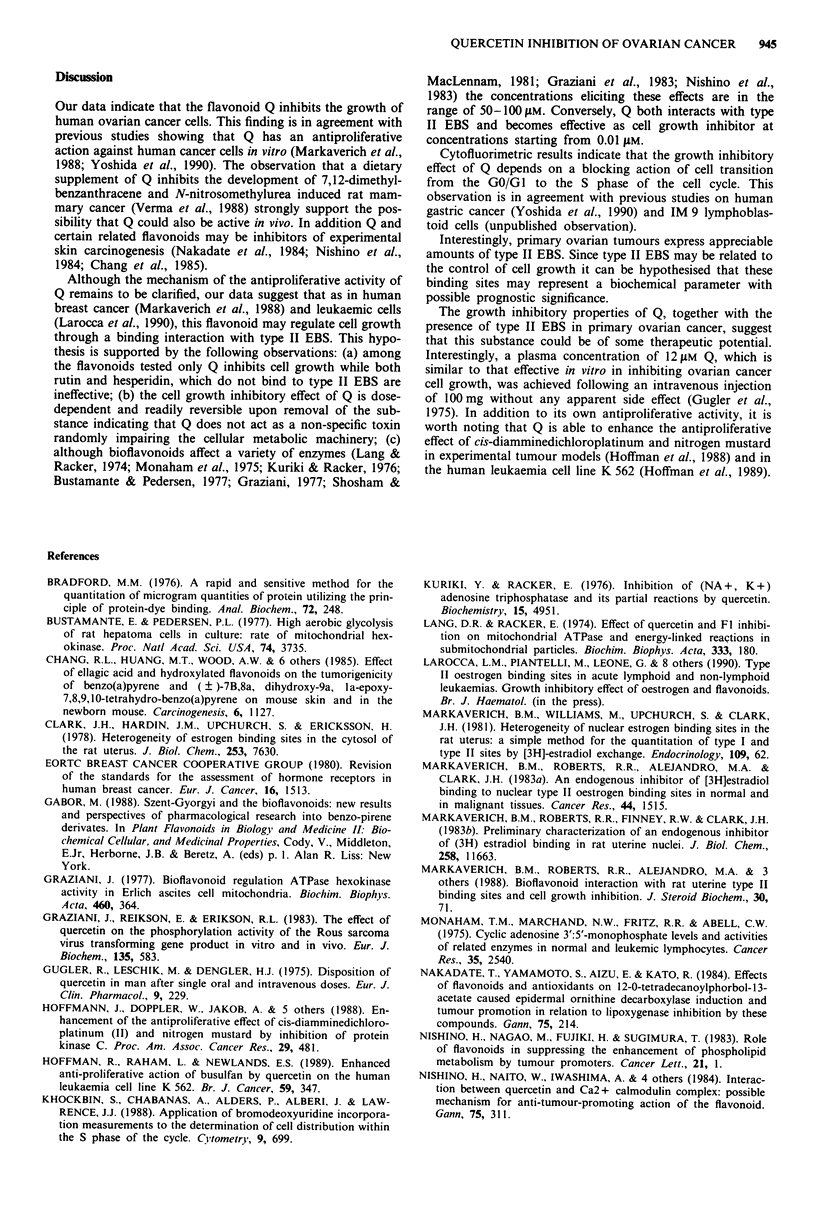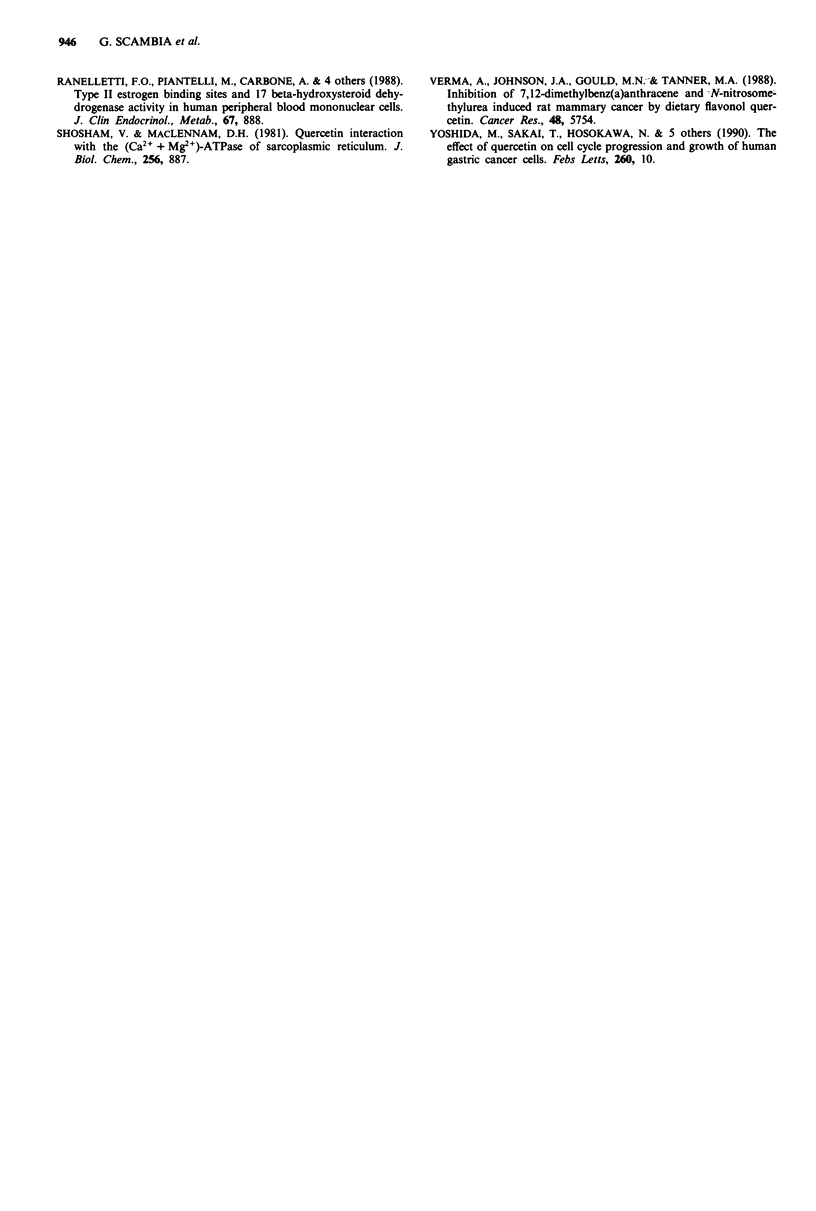# Inhibitory effect of quercetin on OVCA 433 cells and presence of type II oestrogen binding sites in primary ovarian tumours and cultured cells.

**DOI:** 10.1038/bjc.1990.414

**Published:** 1990-12

**Authors:** G. Scambia, F. O. Ranelletti, P. B. Panici, M. Piantelli, G. Bonanno, R. De Vincenzo, G. Ferrandina, C. Rumi, L. M. Larocca, S. Mancuso

**Affiliations:** Department of Gynaecology, Catholic University, Rome, Italy.

## Abstract

We investigated the effect of the flavonoid quercetin (Q) on the proliferation of the ovarian cancer cell line OVCA 433. Growth experiments demonstrated that Q exerted a reversible dose-dependent inhibition of cell proliferation in the range of concentrations between 10 nM and 10 microM. Two other flavonoids tested, rutin and hesperidin, were ineffective in inhibiting cell growth. Cell cycle analysis showed that the growth inhibitory effect of Q was due to a blocking effect in the GO/G1 phase. Using a whole cell assay with (6.7-3H) oestradiol (3H-E2) as tracer we demonstrated that OVCA 433 cells contain type II oestrogen binding sites (type II EBS). Competition analysis showed that Q competed for 3H-E2 binding to type II EBS while both rutin and hesperidin did not. Appreciable amounts of type II EBS were also detected in seven primary ovarian tumours. Our results suggest that Q may regulate ovarian cancer cell growth through a mechanism involving a binding interaction with type II EBS. This mechanism could also be active in vivo since primary ovarian tumours contain type II EBS.


Br. J. Cancer (1990), 62, 942-946 © Macmillan Press Ltd., 1990

### Inhibitory effect of quercetin on OVCA 433 cells and presence of type II oestrogen binding sites in primary ovarian tumours and cultured cells

G. Scambia[1], F.O. Ranelletti[2], P. Benedetti Panici[1], M. Piantelli[3], G. Bonanno[1], R. De Vincenzo[1], G. Ferrandina[1], C. Rumi[4], L.M. Larocca[3] & S. Mancuso[1]

*Departments of [1]Gynaecology, [2]Histology, [3]Pathology and [4]Haematology, Catholic University, Largo A, Gemelli 8, 00168-Rome, Italy.*

**Summary** We investigated the effect of the flavonoid quercetin (Q) on the proliferation of the ovarian cancer cell line OVCA 433. Growth experiments demonstrated that Q exerted a reversible dose-dependent inhibition of cell proliferation in the range of concentrations between 10 nM and 10 μM. Two other flavonoids tested, rutin and hesperidin, were ineffective in inhibiting cell growth. Cell cycle analysis showed that the growth inhibitory effect of Q was due to a blocking effect in the GO/G1 phase. Using a whole cell assay with (6,7-$^3$H) oestradiol ($^3$H-E2) as tracer we demonstrated that OVCA 433 cells contain type II oestrogen binding sites (type II EBS). Competition analysis showed that Q competed for $^3$H-E2 binding to type II EBS while both rutin and hesperidin did not. Appreciable amounts of type II EBS were also detected in seven primary ovarian tumours. Our results suggest that Q may regulate ovarian cancer cell growth through a mechanism involving a binding interaction with type II EBS. This mechanism could also be active *in vivo* since primary ovarian tumours contain type II EBS.

Flavonoids are a widely distributed class of natural substances with a variety of biological actions (Gabor, 1988). Recently it has been reported that in the rat uterus and in the MCF-7 human breast cancer cell line the flavonoid quercetin (Q) inhibits cell growth and the uterotrophic response to oestradiol (Markaverich *et al.*, 1988). Although the mechanism of the antiproliferative activity of Q remains to be fully clarified, there is evidence suggesting that the action of this substance is probably mediated by its interaction with the so-called type II oestrogen binding site (type II EBS) (Markaverich *et al.*, 1988). These sites originally described by Clark *et al.* (1978) in rat uterus, while displaying the same steroid and tissue specificity, are distinct from the 'true' oestrogen receptor (ER). They are reported to be present in higher concentrations than ER but to have a lower apparent affinity dissociation constant ($K_D$ 10-20 nM) for E2 than ER ($K_D$ 0.2-1 nM).

This possibility is supported by the demonstration that in rat uterus type II EBS are occupied *in vivo* by a flavonoid-like ligand with growth inhibitory activity (Markaverich *et al.*, 1983*a,b*). We report here that Q has an antiproliferative effect against human ovarian cancer cells in culture and that these cells express appreciable amounts of type II EBS. Moreover, we demonstrate that type II EBS are also present in primary ovarian tumours.

### Materials and methods

### *Cell culture*

OVCA 433 ovarian cancer cells were kindly provided by Dr B. Littlefield (Department of Gynecology, Yale University, USA). Cells were grown in monolayer culture in minimum essential medium (MEM) supplemented with 10% fetal calf serum (FCS) and 200 units ml$^{-1}$ penicillin. Cells were trypsinised weekly and plated at a density of $8 \times 10^4$ cells ml$^{-1}$. They were incubated at 37°C under 5% $CO_2$ 95% air in a high humidity atmosphere.

Correspondence: S. Mancuso.
Received 18 April 1990; and in revised form 27 June 1990.

### *Growth experiments*

Cells were plated in six-well flat bottom plates (Falcon 3046, Becton Dickinson, Lincoln Park, NJ, USA) at a concentration of $1 \times 10^5$ cells ml$^{-1}$ in MEM supplemented as above. After 24 h, the medium was replaced with fresh medium and Q (3,3',4',5,7-pentahydroxyflavone), rutin (3-rhamnosylglucoside of Q) and hesperidin (7-rhamnosylglucoside of Hesperitin) (3'-5-3-hydroxy-4-methoxy-flavanone) (Aldrich, Steinhein, FRG) were added from an absolute ethanol (Q) or DMSO stock solution (rutin, hesperidin). Control cells were treated with the same amount of vehicle alone. The final ethanol and DMSO concentration never exceeded 1% (v/v) and 0.5% (v/v), in either control or treated samples, respectively.

Quadruplicate haemocytometer counts of triplicate culture dishes were performed at the time indicated in the figures.

### *Cell cycle analysis*

OVCA 433 cells were plated at a concentration of $1 \times 10^5$ cells ml$^{-1}$ in MEM supplemented as above. Twenty-four hours after plating, medium was replaced with fresh medium containing 10 μM Q or vehicle alone (ethanol). After 3 days, cells were incubated for 30 min at 37°C in the same medium with 10 μM bromodeoxyuridine (Sigma Deisenhofen, FRG). Cells were detached with a cell scraper, centrifuged at 1,500 *g* for 5 min at 20°C and washed twice by resuspending in phosphate buffered saline (PBS) pH 7.4 at 20°C. The cell pellet was fixed by resuspending in 50% (v/v) absolute ethanol in PBS containing 10 mM EDTA. Cells were then washed twice with 0.5% (v/v) Tween 20 in PBS and treated with 3 N HCL solution for 30 min at room temperature for DNA denaturation. After two washes with 0.5% (v/v) Tween 20 in PBS the reaction was stopped by resuspending cells with 0.1 M sodium tetraborate pH 8.5. The cell pellet was then resuspended in 100 μl PBS containing 20 μl of anti-bromodeoxy-uridine antibody (Becton Dickinson, Lab Impex Ltd, Middlesex, UK) for 30 min at room temperature. The cells were then washed twice with PBS and incubated for 30 min with 2.5 μl per $10^6$ cells FITC-conjugated goat-anti mouse IgG in PBS. After washing twice with 0.5% (v/v) Tween 20 in PBS the cell pellet was resuspended in PBS containing 25 μg ml$^{-1}$ propidium iodide (Sigma). Stained cells were analysed by the Facscan cytometer (Becton Dickinson) and results calculated according to Khochbin *et al.* (1988).

*Type II EBS analysis in OVCA 433*

Type II EBS were measured with a whole cell assay previously described (Ranelletti *et al.*, 1988) with slight modifications. Cells were plated into Multiwell TM (Falcon 3047) at a concentration of $5 \times 10^4$ cells $ml^{-1}$ in MEM supplemented as above. After 24 h, the medium was replaced with fresh medium without serum containing increasing concentrations (4–50 nM) of $^3$H-E2 (40 Ci $mmol^{-1}$ Amersham, UK) alone or in the presence of a 100-fold molar excess of diethylstilbestrol (DES) (SIGMA) at 4°C for 2.5 h. At the end of the incubation period, cells were rapidly washed twice with ice-cold MEM and then incubated in 1 M NaOH for 30 min at 50°C. Radioactivity was measured by a liquid scintillation spectrometer (LS-7000, Beckman, Palo Alto, CA, USA). Specific binding was calculated as the difference between the binding in the absence (total binding) and in the presence of DES (non-specific binding). Results were expressed as the number of binding sites per cell. The conversion of radioactivity to number of sites per cell was accomplished by determining the amount of $^3$H-E2 bound for aliquots derived from a known number of cells and by applying Avogadro's number.

*Type II EBS analysis in tumour specimens*

Fresh tissue specimens from primary tumours were removed in the operating room, frozen immediately on dry ice, and stored at −80°C until assay. Tissues were finely minced and homogenised in five volumes of ice-cold buffer consisting of 10 mM Tris, 1.5 mM EDTA, 5 mM $NaN_3$ (TEN) by applying three to four 10s bursts of an Ultra-Turrax homogeniser with intermittent cooling. The crude homogenate was centrifuged at 105,000 *g* for 30 min at 0°C and the resulting supernatant was used for type II EBS analysis. Briefly, 250 µl of cytosol were incubated at 30°C for 30 min with increasing concentrations of $^3$H-E2 (4–50 nM) without or with a 300-fold molar excess of DES. Bound and free steroids were separated by hydroxylapatite method, as previously reported (Clark *et al.*, 1978). Specific binding was calculated as the difference between the binding in the absence (total binding) and in the presence (non specific binding) of unlabelled DES. Protein concentration was determined by the method of Bradford (1976).

*Oestrogen and progesterone receptor analysis*

Oestrogen (ER) and progesterone (PR) receptors were assayed according to EORTC protocols (1980).

### Results

*Growth inhibitory effect on Q on OVCA 433 cells*

Figure 1 shows the effects of different flavonoids on OVCA 433 cell proliferation. Q produced a dose-dependent inhibition of cell growth while, even at the highest concentration tested (10 µM), rutin and hesperidin did not have any significant effect. When the time-dependent effect of Q was evaluated, it appeared that the antiproliferative action was already evident 24 h after the addition of the substance (Figure 2).

The inhibitory effect of Q was not due to a non-specific cytotoxic action. In fact, cell viability which was more than 85% did not vary between control and treated cells, after the 3 day culture period. Furthermore, the inhibitory effect appears to be reversible since after the removal of Q, treated OVCA 433 cells regrew like untreated cells (Figure 3).

To study further the antiproliferative activity of Q, we analysed the effect of this substance on the OVCA 433 cell cycle. As shown in Table I, the cytometric flow analysis revealed that Q produces an increase in the percentage of cells in the G0/G1 phase of the cycle with a relative decrease of those in the S phase.

**Figure 1** Effect of various concentrations of flavonoids on OVCA 433 cell proliferation. Cell counts were performed after 3 days of exposure to quercetin (●), rutin (□) and hesperidin (Δ). Each value represents the mean ± s.d. of three different experiments performed in triplicate.

**Figure 2** Time course of the antiproliferative effect of quercetin (Q) on OVCA 433 cells. Cells were cultured without (O) or with Q (●) at 10 µM, for the time indicated. Results represent the means ± s.d. of triplicate determinations from one of two similar experiments.

**Table I** Effect of quercetin on the distribution of OVCA 433 cells in the different stages of the cell cycle

| | Treatment[a] | |
|---|---|---|
| | *None* | *Q (10 µM)* |
| G0/G1 | 68.15 ± 2.10[b] | 78.24 ± 2.54[c] |
| S | 26.73 ± 0.68 | 16.56 ± 0.49[c] |
| G2/M | 5.1 ± 0.13 | 5.1 ± 0.21 |

[a]OVCA 433 cells were cultured for two days without or with Q at 10 µM; [b]Results are expressed as percentage of cells in each phase of the cell cycle. Values represent the means ± s.d. of triplicate determinations from one of three similar experiments; [c]$P < 0.01$ compared with untreated cells by Student's *t* test.

**Figure 3** Reversibility of the antiproliferative effect of quercetin (Q) on OVCA 433 cells. Cells were cultured without (○) or with 10 μM Q (●) for 2 days or continuously (dotted line). Results represent the means ± s.d. of triplicate determinations from one of two similar experiments.

**Figure 4** **a**, Specific binding of $^3$H-E2 as a function of tracer concentration in OVCA 433 cells. Specifically bound $^3$H-E2 was measured as detailed in Materials and methods. **b**, Scatchard analysis of data from **a**.

**Table II** Steroid specificity of type II EBS in OVCA 433

| *Competing steroids*[a] | *% binding* |
|---|---|
| None | 100 |
| 17-β-oestradiol | 37 ± 6 |
| Diethylstilbestrol | 32 ± 7 |
| Progesterone | 99 ± 12 |
| 5-α-dihydrotestosterone | 100 ± 13 |
| Androstenedione | 95 ± 10 |
| Dexamethasone | 90 ± 8 |
| Quercetin | 25 ± 8 |
| Rutin | 88 ± 15 |
| Hesperidin | 95 ± 8 |

Values are the mean ± s.d. ($n = 5$ different experiments). [a]All competing steroids were at a 100-fold molar excess relative to $^3$H-E2 (40 nM).

*Type II EBS in OVCA 433 cells*

Since Q has been reported to exert its inhibitory effect through an interaction with type II EBS (Markaverich *et al.*, 1988) we looked for the presence of these binding sites in OVCA 433 cells. As shown in Figure 4a, in these cells the saturation analysis carried out by a whole cell assay at 4°C for 2.5 h resulted in a sigmoid curve with saturation occurring between 40 and 50 nM $^3$H-E2. As predicted from the biphasic nature of the saturation curve, Scatchard analysis of the binding values yielded a concave plot (Figure 4b) similar to that previously observed in other type II expressing systems (Ranelletti *et al.*, 1988). Since an accurate estimate of both the $K_D$ and the number of EBS cannot be made from a curvilinear Scatchard plot, these parameters were obtained from the saturation curve (Clark *et al.*, 1978; Ranelletti *et al.*, 1988).

For the experiment shown in Figure 4 the number of type II EBS calculated from the saturation curve at maximum binding is about 290,000 sites per cell. The $K_D$ determined from the $^3$H-E2 concentration required for half saturation is about 18 nM. In six similar experiments the number of sites per cell and the $K_D$ values were 320,900 ± 85,000 s.d. and 16 nM ± 3 s.d., respectively. Specificity experiments demonstrated that among different steroids tested only oestrogenic compounds inhibited the binding of $^3$H-E2 to type II EBS (Table II). The data in the same table show that Q is able to compete with $^3$H-E2 for type II EBS binding with a potency similar to that of DES. Furthermore, 3-rhamnosyl-glucoside of Q (rutin) and hesperetin-7-rutinoside (hesperidin) do not compete at all.

*Type II EBS in primary ovarian cancer*

Type II EBS are expressed in appreciable amounts in primary ovarian tumours too. The morphology of both $^3$H-E2 binding curve to type II EBS and the Scatchard plot of binding data closely resemble those observed for OVCA 433 cells (data not shown). As shown in Table III all tumour specimens tested contained type II EBS, with values ranging from 2665 fmol mg$^{-1}$ of protein to 5,200 fmol mg$^{-1}$ protein and $K_D$ values from 13 to 18 nM. In the presence of 10 mM dithiothreitol (DTT) the binding of $^3$H-E2 to type II EBS was reduced to approximately 30% of the control (without DTT) value (data not shown). This sensitivity to reducing agents is similar to that previously observed for type II EBS in rat uterus (Markaverich *et al.*, 1981).

In all cases Q displaced $^3$H-E2 from the type II EBS with a potency similar to that of DES, since the percentage of displacement are between 80% and 90% of the total bound. Four and three cases expressed ER and PR respectively (Table III). However, although the number of specimens tested are very small, no correlation was found between type II EBS levels and ER and PR concentration. Furthermore, Q did not compete for $^3$H-E2 binding to ER (data not shown).

**Table III** Type II EBS ER and PR in primary ovarian tumours

| *Patient* | *Histology* | *Type II* | *EBS ($K_D$, nM)* | *ER ($K_D$, nM)* | *PR ($K_D$, nM)* |
|---|---|---|---|---|---|
| A.T. | Serous | 4875[a] | (13)[b] | 55 (0.6)[c] | 120 (0.8) |
| V.P. | Serous | 4943 | (15) | n.d. | n.d. |
| M.G.R. | Serous | 5200 | (14) | 12 (1) | 18 (0.4) |
| O.M. | Serous | 3569 | (18) | n.d. | n.d. |
| P.M. | Serous | 2665 | (16) | 26 (0.4) | n.d. |
| L.M. | Serous | 4191 | (15) | n.d. | n.d. |
| G.C. | Serous | 3492 | (14) | 66 (0.6) | 44 (0.7) |

n.d. = not detectable. [a]fmol mg$^{-1}$ of protein calculated from the saturation curve at maximum binding. [b]Calculated as the $^3$H-E2 concentration required for half-saturation. [c]fmol mg$^{-1}$ of protein and ($K_D$) calculated from the Scatchard's analysis of binding data.

### Discussion

Our data indicate that the flavonoid Q inhibits the growth of human ovarian cancer cells. This finding is in agreement with previous studies showing that Q has an antiproliferative action against human cancer cells *in vitro* (Markaverich *et al.*, 1988; Yoshida *et al.*, 1990). The observation that a dietary supplement of Q inhibits the development of 7,12-dimethylbenzanthracene and *N*-nitrosomethylurea induced rat mammary cancer (Verma *et al.*, 1988) strongly support the possibility that Q could also be active *in vivo*. In addition Q and certain related flavonoids may be inhibitors of experimental skin carcinogenesis (Nakadate *et al.*, 1984; Nishino *et al.*, 1984; Chang *et al.*, 1985).

Although the mechanism of the antiproliferative activity of Q remains to be clarified, our data suggest that as in human breast cancer (Markaverich *et al.*, 1988) and leukaemic cells (Larocca *et al.*, 1990), this flavonoid may regulate cell growth through a binding interaction with type II EBS. This hypothesis is supported by the following observations: (a) among the flavonoids tested only Q inhibits cell growth while both rutin and hesperidin, which do not bind to type II EBS are ineffective; (b) the cell growth inhibitory effect of Q is dose-dependent and readily reversible upon removal of the substance indicating that Q does not act as a non-specific toxin randomly impairing the cellular metabolic machinery; (c) although bioflavonoids affect a variety of enzymes (Lang & Racker, 1974; Monaham *et al.*, 1975; Kuriki & Racker, 1976; Bustamante & Pedersen, 1977; Graziani, 1977; Shosham & MacLennam, 1981; Graziani *et al.*, 1983; Nishino *et al.*, 1983) the concentrations eliciting these effects are in the range of 50–100 μM. Conversely, Q both interacts with type II EBS and becomes effective as cell growth inhibitor at concentrations starting from 0.01 μM.

Cytofluorimetric results indicate that the growth inhibitory effect of Q depends on a blocking action of cell transition from the G0/G1 to the S phase of the cell cycle. This observation is in agreement with previous studies on human gastric cancer (Yoshida *et al.*, 1990) and IM 9 lymphoblastoid cells (unpublished observation).

Interestingly, primary ovarian tumours express appreciable amounts of type II EBS. Since type II EBS may be related to the control of cell growth it can be hypothesised that these binding sites may represent a biochemical parameter with possible prognostic significance.

The growth inhibitory properties of Q, together with the presence of type II EBS in primary ovarian cancer, suggest that this substance could be of some therapeutic potential. Interestingly, a plasma concentration of 12 μM Q, which is similar to that effective *in vitro* in inhibiting ovarian cancer cell growth, was achieved following an intravenous injection of 100 mg without any apparent side effect (Gugler *et al.*, 1975). In addition to its own antiproliferative activity, it is worth noting that Q is able to enhance the antiproliferative effect of *cis*-diamminedichloroplatinum and nitrogen mustard in experimental tumour models (Hoffman *et al.*, 1988) and in the human leukaemia cell line K 562 (Hoffman *et al.*, 1989).

### References

BRADFORD, M.M. (1976). A rapid and sensitive method for the quantitation of microgram quantities of protein utilizing the principle of protein-dye binding. *Anal. Biochem.*, **72**, 248.

BUSTAMANTE, E. & PEDERSEN, P.L. (1977). High aerobic glycolysis of rat hepatoma cells in culture: rate of mitochondrial hexokinase. *Proc. Natl Acad. Sci. USA*, **74**, 3735.

CHANG, R.L., HUANG, M.T., WOOD, A.W. & 6 others (1985). Effect of ellagic acid and hydroxylated flavonoids on the tumorigenicity of benzo(a)pyrene and (±)-7B,8a, dihydroxy-9a, 1a-epoxy-7,8,9,10-tetrahydro-benzo(a)pyrene on mouse skin and in the newborn mouse. *Carcinogenesis*, **6**, 1127.

CLARK, J.H., HARDIN, J.M., UPCHURCH, S. & ERICKSSON, H. (1978). Heterogeneity of estrogen binding sites in the cytosol of the rat uterus. *J. Biol. Chem.*, **253**, 7630.

EORTC BREAST CANCER COOPERATIVE GROUP (1980). Revision of the standards for the assessment of hormone receptors in human breast cancer. *Eur. J. Cancer*, **16**, 1513.

GABOR, M. (1988). Szent-Gyorgyi and the bioflavonoids: new results and perspectives of pharmacological research into benzo-pirene derivates. In *Plant Flavonoids in Biology and Medicine II: Biochemical Cellular, and Medicinal Properties*, Cody, V., Middleton, E.Jr, Herborne, J.B. & Beretz, A. (eds) p. 1. Alan R. Liss: New York.

GRAZIANI, J. (1977). Bioflavonoid regulation ATPase hexokinase activity in Erlich ascites cell mitochondria. *Biochim. Biophys. Acta*, **460**, 364.

GRAZIANI, J., REIKSON, E. & ERIKSON, R.L. (1983). The effect of quercetin on the phosphorylation activity of the Rous sarcoma virus transforming gene product in vitro and in vivo. *Eur. J. Biochem.*, **135**, 583.

GUGLER, R., LESCHIK, M. & DENGLER, H.J. (1975). Disposition of quercetin in man after single oral and intravenous doses. *Eur. J. Clin. Pharmacol.*, **9**, 229.

HOFFMANN, J., DOPPLER, W., JAKOB, A. & 5 others (1988). Enhancement of the antiproliferative effect of cis-diamminedichloroplatinum (II) and nitrogen mustard by inhibition of protein kinase C. *Proc. Am. Assoc. Cancer Res.*, **29**, 481.

HOFFMAN, R., RAHAM, L. & NEWLANDS, E.S. (1989). Enhanced anti-proliferative action of busulfan by quercetin on the human leukaemia cell line K 562. *Br. J. Cancer*, **59**, 347.

KHOCKBIN, S., CHABANAS, A., ALDERS, P., ALBERI, J. & LAWRENCE, J.J. (1988). Application of bromodeoxyuridine incorporation measurements to the determination of cell distribution within the S phase of the cycle. *Cytometry*, **9**, 699.

KURIKI, Y. & RACKER, E. (1976). Inhibition of (NA+, K+) adenosine triphosphatase and its partial reactions by quercetin. *Biochemistry*, **15**, 4951.

LANG, D.R. & RACKER, E. (1974). Effect of quercetin and F1 inhibition on mitochondrial ATPase and energy-linked reactions in submitochondrial particles. *Biochim. Biophys. Acta*, **333**, 180.

LAROCCA, L.M., PIANTELLI, M., LEONE, G. & 8 others (1990). Type II oestrogen binding sites in acute lymphoid and non-lymphoid leukaemias. Growth inhibitory effect of oestrogen and flavonoids. *Br. J. Haematol.* (in the press).

MARKAVERICH, B.M., WILLIAMS, M., UPCHURCH, S. & CLARK, J.H. (1981). Heterogeneity of nuclear estrogen binding sites in the rat uterus: a simple method for the quantitation of type I and type II sites by [3H]-estradiol exchange. *Endocrinology*, **109**, 62.

MARKAVERICH, B.M., ROBERTS, R.R., ALEJANDRO, M.A. & CLARK, J.H. (1983*a*). An endogenous inhibitor of [3H]estradiol binding to nuclear type II oestrogen binding sites in normal and in malignant tissues. *Cancer Res.*, **44**, 1515.

MARKAVERICH, B.M., ROBERTS, R.R., FINNEY, R.W. & CLARK, J.H. (1983*b*). Preliminary characterization of an endogenous inhibitor of (3H) estradiol binding in rat uterine nuclei. *J. Biol. Chem.*, **258**, 11663.

MARKAVERICH, B.M., ROBERTS, R.R., ALEJANDRO, M.A. & 3 others (1988). Bioflavonoid interaction with rat uterine type II binding sites and cell growth inhibition. *J. Steroid Biochem.*, **30**, 71.

MONAHAM, T.M., MARCHAND, N.W., FRITZ, R.R. & ABELL, C.W. (1975). Cyclic adenosine 3′:5′-monophosphate levels and activities of related enzymes in normal and leukemic lymphocytes. *Cancer Res.*, **35**, 2540.

NAKADATE, T., YAMAMOTO, S., AIZU, E. & KATO, R. (1984). Effects of flavonoids and antioxidants on 12-0-tetradecanoylphorbol-13-acetate caused epidermal ornithine decarboxylase induction and tumour promotion in relation to lipoxygenase inhibition by these compounds. *Gann*, **75**, 214.

NISHINO, H., NAGAO, M., FUJIKI, H. & SUGIMURA, T. (1983). Role of flavonoids in suppressing the enhancement of phospholipid metabolism by tumour promoters. *Cancer Lett.*, **21**, 1.

NISHINO, H., NAITO, W., IWASHIMA, A. & 4 others (1984). Interaction between quercetin and Ca2+ calmodulin complex: possible mechanism for anti-tumour-promoting action of the flavonoid. *Gann*, **75**, 311.

RANELLETTI, F.O., PIANTELLI, M., CARBONE, A. & 4 others (1988). Type II estrogen binding sites and 17 beta-hydroxysteroid dehydrogenase activity in human peripheral blood mononuclear cells. *J. Clin Endocrinol., Metab.*, **67**, 888.

SHOSHAM, V. & MACLENNAM, D.H. (1981). Quercetin interaction with the ($Ca^{2+}$ + $Mg^{2+}$)-ATPase of sarcoplasmic reticulum. *J. Biol. Chem.*, **256**, 887.

VERMA, A., JOHNSON, J.A., GOULD, M.N. & TANNER, M.A. (1988). Inhibition of 7,12-dimethylbenz(a)anthracene and *N*-nitrosomethylurea induced rat mammary cancer by dietary flavonol quercetin. *Cancer Res.*, **48**, 5754.

YOSHIDA, M., SAKAI, T., HOSOKAWA, N. & 5 others (1990). The effect of quercetin on cell cycle progression and growth of human gastric cancer cells. *Febs Letts*, **260**, 10.